# Patterns of exclusive, dual and poly tobacco products use among cancer survivors in the United States

**DOI:** 10.1007/s11764-025-01918-x

**Published:** 2025-12-16

**Authors:** Ateeqa Ijaz, Miranda E. Gehris, Paul T. Harrell, Aditya Chakraborty, Rime Jebai, Wei Li, Olatokunbo Osibogun, Seyedeh Yasaman Alemohammad, Ziyad Ben Taleb, Ebbie Kalan

**Affiliations:** 1 School of Health Professions, Macon & Joan Brock Virginia Health Sciences at Old Dominion University, Norfolk, VA USA; 2 Montana Department of Corrections, Central Services Division - Research, Analytics, & Planning, Helena, USA; 3 Department of Pediatrics, Macon & Joan Brock Virginia Health Sciences at Old Dominion University, Norfolk, VA USA; 4 Department of Epidemiology, Biostatistics & Environmental Health, Joint School of Public Health, Old Dominion University, Norfolk, VA USA; 5 Department of Health Law, Policy & Management, Boston University, Boston, MA USA; 6 Department of Psychiatry, Yale School of Medicine, New Haven, CT USA; 7 Department of Epidemiology, Robert Stempel College of Public Health, Florida International University, Miami, FL USA; 8 Department of Kinesiology, College of Nursing and Health Innovation, University of Texas at Arlington, Arlington, TX USA; 9 Department of Behavioral and Community Health, School of Public Health, University of Maryland, 4200 Valley Dr, College Park, MD USA

**Keywords:** Cancer, Survivors, Tobacco, Cigarettes, E-cigarettes, Dual use, Poly-use, Tobacco-related cancers

## Abstract

**Purpose:**

Identifying tobacco products use (TPU) patterns among cancer survivors (CSs) is critical to optimize survivorship care. This study examines exclusive cigarette/e-cigarette, dual (two tobacco-products) and poly (≥ 3 tobacco-products) use among CSs (vs. no cancer history) and its sub-types: smoking-related-cancers (SRCs) vs. non-smoking-related cancers (NSRCs) while also exploring socio-demographic factors.

**Methods:**

The study analyzed 86,551 adults from the 2021–2023 National Health Interview Survey, including 10,846 (9.7%) CSs. Participants were categorized and analyzed based on their CSs status and TPU patterns. Weighted logistic regression models assessed the relationship between TPU and CSs status while adjusting for socio-demographic characteristics.

**Results:**

CSs (vs. no cancer history) had a higher likelihood of ever-poly (aOR:1.24, 95%CI: 1.15–1.33), former-cigarette-only (aOR:1.22, 95%CI: 1.13–1.32), and former-dual (aOR:1.21, 95%CI: 1.14–1.29) use. SRC survivors had greater odds of being current-cigarette-only (aOR:2.26, 95%CI: 1.63–3.11), ever e-cigarette-only (aOR:3.41, 95%CI: 1.51–7.70), and ever-poly (aOR:1.32, 95%CI: 1.11–1.57) use than NSRC survivors. Among CSs, being male, single after marriage, or having lower educational attainment was linked to higher odds of exclusive, dual, or poly-tobacco use. CSs aged over 44 years had lower odds of being dual or ever-poly users compared to younger adults.

**Conclusion:**

Disparities in TPU among CSs particularly between SRC and NSRC survivors calls for proactive cessation support. Oncologists must account for patients’ former and current TPU pattern, related perceptions, and socio-demographic barriers in cessation by systematically incorporating tailored and integrated counseling into routine oncology visits.

**Implications for Cancer Survivors:**

Persistent disparities in tobacco product use requires tailored cessation support in routine oncology visits.

**Supplementary Information:**

The online version contains supplementary material available at https://doi.org/10.1007/s11764-025-01918-x.

## Introduction

Cancer survivors (CSs), who constitute approximately 5.4% of the US population in 2022, are projected to increase by 24.4% by 2030, reflecting an extended survival length [1, 2]. As the post-diagnosis survival length increases, it becomes increasingly critical to monitor their health behaviors, particularly smoking [1]. The US Surgeon General’s Report on smoking has eloquently detailed the causal role of cigarette smoking and its adverse health effects, yet 10 to 20% of US. CSs are current cigarette smokers [3]. Moreover, the National Cancer Institute’s report (NCI-2022) shows a sequential increase in the prevalence of cigarette smoking with each year of initial cancer diagnosis [2]. Additionally, 33% of CSs who had successfully quit smoking relapsed. Continued cigarette smoking among CSs raises the risk of poorer oncological outcomes, including a higher chance of second primary malignancies, increase the risk of overall mortality, and cancer-specific mortality and morbidities [4, 5]. In addition, it reduces the effectiveness of treatment modalities such as chemotherapy, radiation therapy, and immunotherapy by causing changes in tumor biology [4, 6]. Hence, monitoring smoking behaviors serves as a pivotal tool in evaluating the effectiveness of ongoing cessation efforts to optimize the cancer survivorship journey and assist in reducing preventable deaths [1].

Although cigarette smoking remains the predominant form of nicotine consumption among CSs, e-cigarette use and dual use (both cigarettes and e-cigarette use) have also been reported [4, 7]. The shift of concurrently using both e-cigarette and cigarette among CSs is prominent and is mainly due to the cessation properties of e-cigarettes and being recommended as a less harmful alternative to conventional smoking by providers [4, 8–10]. A systematic review detailed that more than two-third of the CSs are concurrent users of both traditional cigarette and e-cigarettes with 75% of them reported its use as quit resource [11]. However, there is limited and conflicting evidence regarding its harm reduction strategy and health impact in oncology settings. CSs rarely discuss the concurrent use of e-cigarettes with cigarettes with their oncology providers [12]. This and the former mentioned reasoning could have played a critical role in stagnant and less effective cessation programs among CSs than the general population as in 2022, approximately 11.4% of CSs in the US were current cigarette smokers, a rate consistent with the 11.5% in 2017 [13].

In addition to cigarettes, e-cigarettes, and their concurrent use, poly tobacco product use (TPU) has also been reported among CSs. A study in 2019 reported that among smokers, 6.9% of CSs were poly-tobacco users, and 25% were dual users [4]. Thus, TPU behaviors among CSs is not only confined to cigarette or e-cigarette use, but also to alternative tobacco products which are playing a critical role in hindering the cessation efforts among CSs. Furthermore, these TPU behaviors varies by CSs subtypes (i.e., Smoking Related Cancer (SRC) and Non-Smoking Related Cancer (NSRC) survivors). A recent study in 2022 reported that the SRC survivors have higher odds of being current cigarette smokers than NSRC survivors [14, 15]. Additionally, about 18.2% of SRC survivors in the U.S. currently smoke cigarettes, and 2.7% use e-cigarettes, while 9.7% of NSRC survivors smoke cigarettes and 1.6% use e-cigarettes with less being known about the poly-tobacco use among them [14]. These TPU behaviors also varies across socio-demographics groups with young adult CSs (aged 18–44) were reported to have higher rates of current cigarette and e-cigarette users than other age groups [4]. Henceforth, comparative analysis to examine the patterns of TPU (mono, dual, and poly) between cancer sub-types (SRCs vs NSRCs) is essential because SRC survivors differs in their nicotine dependence levels, smoking histories, and cessation motivations compared to NSRC survivors [16, 17]. For example, SRC survivors often have established tobacco use that directly contributed to their diagnosis, while NSRC survivors developed tobacco use independently of their cancer. Moreover, both groups experience different symptoms burden: pain, fatigue, depression, and anxiety, which influence tobacco use and relapse patterns [16, 18].

Although previous studies have extensively examined cigarettes, e-cigarettes, and dual use among CSs and compared it with general population [4, 9, 10, 12, 19–21], there has been limited research on poly TPU and the comparative analysis of TPU behaviors among CSs subtypes (SRCs vs NSRCs) [7, 14, 15, 19, 21]. Importantly, they lacked analysis of mutually exclusive categories for TPU which often complicates the understanding of TPU by product and limits the ability to design targeted public health interventions. To address this gap, our study classified participants into mutually exclusive TPU groups, enabling its detailed comparisons for CSs stratified by cancer type i.e., SRCs vs. NSRCs and with adults without a cancer history. This comparison enables identification of CSs subtype-specific cessation needs and barriers, supporting tailored interventions that can improve long-term health outcomes for each population [22, 23]. We also provided detailed insights into dual use by reporting all possible TPU combinations and examining key demographic differences within the CSs. Together, these contributions offer a clearer epidemiological understanding of TPU and support the development of more precise and effective cessation strategies for this vulnerable population.

## Materials and methods

### Data source and study participants

The National Health Interview Survey (NHIS) is an annual cross-sectional study monitoring US population health. NHIS employs a complex weighting procedure, which includes sampling probabilities, adjustment for non-response, and post-stratification to the US population benchmarks, to ensure the sample is representative of US adults [24]. This study analyzed the publicly available de-identified NHIS data from 2021 to 2023 involving adults aged ≥ 18 years (N = 86,657). It included participants who responded to the question about their cancer diagnosis status (N = 86,551) and excluded those who did not report or were missing a response (n = 104) from the analytical sample (see Online Resource I: study sample flow-chart for details). The NHIS survey composition and research strategy can be found elsewhere [25].

### Cancer status

This study participants were classified into two main categories: “no cancer history” and “Cancer Survivors (CSs).” If they responded “Yes” to the question: “Have you ever been told by a doctor or other health professional that you had cancer or a malignancy of any kind?” they were classified as CSs (n = 10,846), and those with “No” responses classified as the population with no cancer history (n = 75,705). CSs were further classified as smoking-related cancer (SRC) survivors (n = 1,743) and non-smoking-related cancer (NSRC) survivors (n = 8,170). SRCs include cancers of the bladder, cervix, colon, esophagus, kidney, larynx, liver, lung, mouth, pancreas, rectum, stomach, and throat while NSRCs include cancers of the bone, brain, breast, gallbladder, lymphoma, melanoma, nervous system, ovary, prostate, skin, soft tissue, testis, and uterine [14, 26].

### Assessment of tobacco use patterns: exclusive, dual, and poly use

The Tobacco Product Use (TPU) patterns were assessed for: exclusive use (consumption of only one tobacco product without reported use of other tobacco products [OTPs] including cigar/cigarillos/pipe/smokeless tobacco]), dual use (concurrent use of two tobacco products), and poly use (use of three or more tobacco products) [27].

#### Exclusive, dual and poly use

The exclusive use was assessed for cigarette and e-cigarettes and was categorized into five mutually exclusive categories. The categories were: never users of any of the tobacco products (coded as 0), current cigarette-only users (1), former cigarette-only (2), current e-cigarette-only (3), and former e-cigarette-only (4). Exclusive current cigarette-only users were those reporting current cigarette use but no e-cigarettes/OTPs while former cigarette-only users were those who reported former cigarette-only and no e-cigarette/OPTs use. A similar strategy was used to define current e-cigarette-only and former e-cigarette-only users’ categories.

**Fig. 1:**
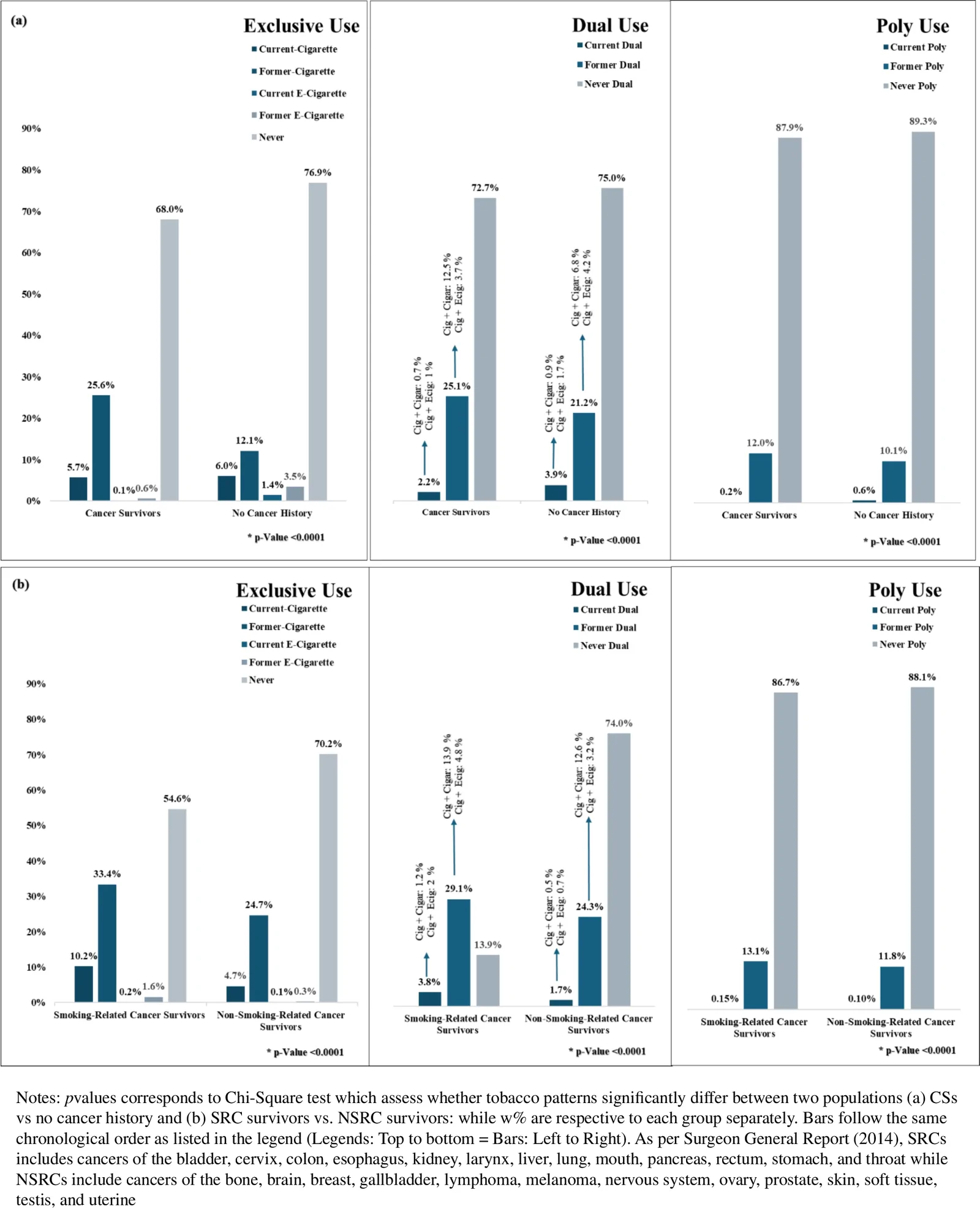
Tobacco Product Use Pattern among US adults by (**a**) Cancer Status (**b**) Cancer Survivors Sub-types, NHIS (2021–2023)

Dual use and poly use were categorized at three levels: current, former, and never users. For dual use ten possible dual use combinations were derived by pairing cigarettes, e-cigarettes, and OTPs. Those who endorsed any dual combination were labeled as “dual users.” All combinations were also analyzed separately (see Online Resource IV and V), but only the most prevalent were reported in the main text (Fig. 1). The poly use was measured by assessing the number of tobacco products used. Those who reported concurrent use of three or more tobacco products (cigarettes, e-cigarettes, or OTPs) were classified as poly users.

To examine the role of e-cigarettes as a cessation aid, NHIS data from 2021–2023 were reviewed; only the 2022 survey included relevant questions. Former smokers were asked: “Thinking back to when you stopped smoking completely, did you try to quit by switching to electronic or e-cigarettes?” while current smokers were asked “Thinking back to when you tried to QUIT smoking in the PAST 12 MONTHS, did you use ANY of the following products: Quit method-e-cigarettes, attempted to quit smoking.” However, due to the limited sample size of CSs responded to these questions in a single survey year, we were unable to conduct a comprehensive inferential analysis though we explored the descriptive analysis (see Results and Online Resource VI).

For OTP use status, two NHIS questions were utilized to measure current, former, and never use: “Have ever smoked a regular cigar, cigarillo, or a little filtered cigar EVEN ONE TIME?” and “if they NOW smoke regular cigars, cigarillos, or little filtered cigars every day, some days, or not at all?” Participants who answered “yes” to ever-use and reported smoking every day or some days were classified as “current” users. Those who answered “yes” to ever-use but reported “not at all” to current use were classified as “former” users. Participants who did not report ever-use were classified as “never” users.

Lastly, current, former, and never status categories in the study followed NHIS definitions: participants who reported smoking at least 100 cigarettes in their lifetime and were currently smoking every day or some days were categorized as current-cigarette smokers. Those who reported smoking at least 100 cigarettes in their lifetime but currently “not smoking at all” were categorized as former-cigarette smokers, while those who reported neversmoking 100 cigarettes and were currently not smoking were categorized as never-cigarette smokers [15]. A similar approach was employed to categorize e-cigarette use as current, former, or never, using the questions: “Have you ever used an e-cigarette or other electronic vaping product, even just one time, in your entire life?” and “Do you now use e-cigarettes or other electronic vaping products every day, some days, or not at all?” [15, 28].

### Socio-demographic

The socio-demographic factors included age (18–44 years, 45–64 years, and 65 and above), sex (male/female), race/ethnicity (Hispanic, non-Hispanic White, non-Hispanic Black, non-Hispanic Others), education (GED or less, high-school, some college, associate or bachelor’s degree and master’s and above), sexual-orientation (Lesbian/gay, bisexual, straight), marital status (married/never married/single after marriage), employment status (Yes/No), poverty status (poor, near poor, and not poor), insurance coverage (Yes/No) and US census region (Northeast, Midwest, South, and West). These socio-demographic factors were included for robust analysis and to identify disparities in TPU among CSs.

Alongside socio-demographic factors, the study controlled for depression due to its high prevalence among CSs and it being reported as a risk factor for the use of various tobacco products [24, 29–34]. Therefore, controlling was necessary to isolate the unbiased relationship between CSs status and TPU. Depression was assessed based on the NHIS question: *Do you take prescription medication for depression?* (Yes/No)*.*

### Statistical analysis

Data from the year 2021 to 2023 of NHIS were analyzed using SAS (version 9.4). All analysis included the proposed survey weight variables to account for complex survey design and ensure nationally representative estimates. Descriptive analyses were utilized to report socio-demographic characteristics, TPU (exclusive use, dual use, poly use), and depression for cancer status and CSs sub-types as frequencies (n), weighted percentages (w%), along with their 95% confidence intervals (CIs). Chi-Square assessed differences in TPU by cancer status and sub-types, with significance at *p* < 0.05.

The association between TPU with cancer status and sub-types, was initially examined using weighted regression analysis adjusted for socio-demographic factors and depression. In regression analysis: the current and former user categories of exclusive e-cigarette-use and poly use were combined into ever e-cigarette users, and ever-poly users, respectively. This “ever use” classification was applied to address the small sample size in current and former categories, which improved model stability and allowed more reliable comparison between ever-users and never-users. Multinomial regression analysis measured the exclusive and dual tobacco use while binary regression was utilized to measure the poly use (ever-user vs. never-users).

#### Stratification analysis

To examine socio-demographic factors associated with each TPU separate regression analysis with only CSs as the analysis sample was conducted. The models included all socio-demographic factors as independent variables. Multinomial regression analysis was used for exclusive and dual use of tobacco products while binary regression analysis was applied to ever-poly use due to its binary nature. Adjusted odds ratios (aORs) and 95% CI were reported for all models. Statistical significance was defined as *p* < 0.05. To address small cell counts and avoid non-convergence issues, race and ethnicity were recoded as “non-Hispanic (all races)” and “Hispanic,” as shown in Table 2.

## Results

### Sample characteristics

As shown in Table 1, in 2023, 9.7% (approximately 28 million) of US adults were CSs and 18.4% of them were classified as SRC survivors. The sociodemographic characteristics of adults with no cancer history and those who were CSs [overall] were mostly similar, with an exception in their age, educational attainment, and sexual orientation. Both groups (CSs and no cancer history) had a majority of the individuals who were non-Hispanic White, females, married, had healthcare coverage, and were not poor. However, most of the CSs [overall] were older (≥ 65 years), had high school or less educational attainment, and few of them were identified as bisexual compared to adults with no cancer history. SRC survivors also had similar characteristics as NSRC survivors: both had a higher proportion of straight, married, not poor, female, and older adults (≥ 65 years) with exceptions in race/ethnicity and educational attainment. However, they varied as SRC survivors had Hispanics as second prominent ethnicity while NSRC survivors had non-Hispanic Black.

**Table 1:** Characteristics of US Adults (N = 86,551)*: CSs, SRC survivors, NSRC survivors and adults* with no Cancer History, NHIS, 2021–2023^*a*^

Characteristics	Cancer Survivors^b^						No Cancer History	
	**Overall**		**SRC Survivors**		**NSRC Survivors**			
	**N (w%)**	**95% CI**	**N (w%)**	**95% CI**	**N (w%)**	**95% CI**	**N (w%)**	**95% CI**
	10,846 (9.68)	9.48–9.88	1743 (18.44)	17.53–19.34*	8170 (81.56)	80.66–82.47	75,705 (90.32)	90.12–90.52
**Age (in years)**								
18–44	721 (9.41)	8.67–10.14	186 (14.47)	12.27–16.67	428 (7.32)	6.58–8.05	30,874 (49.60)	49.18–50.02
45–64	2784 (31.27)	30.21–32.33	504 (34.06)	31.39–36.72	1980 (29.95)	28.74–31.15	24,681 (31.93)	31.54–32.31
65 and above	7341 (59.32)	58.22–60.43	1053 (51.48)	48.72–54.23	5762 (62.74)	61.48–23.99	20,150 (18.48)	18.19–18.76
**Biological Sex**								
Male	4611 (44.56)	43.48–45.65	748 (45.17)	42.43–47.91	3494 (44.48)	43.24–45.72	34,769 (49.02)	48.60–49.44
Female	6235 (55.44)	54.35–56.52	995 (54.83)	52.09–57.57	4676 (55.52)	54.28–56.76	40,925 (50.98)	50.56–51.40
**Marital Status**								
Never Married	5065 (32.36)	34.25–36.26	885 (40.74)	38.07–43.42	3702 (33.41)	32.29–34.53	34,052 (40.16)	39.74–40.59
Married	5135 (60.41)	59.36–61.45	728 (54.40)	51.64–57.15	4031 (62.53)	61.36–63.71	33,472 (50.61)	50.18–51.04
Single After Marriage	333 (4.34)	3.85–4.82	59 (4.86)	3.58–6.14	238 (4.06)	3.52–4.60	5156 (9.22)	8.96–9.49
**Education level**								
High School or less	3848 (34.01)	32.99–35.03	725 (44.59)	41.84–47.35	2318 (30.65)	29.47–31.83	27,314 (33.60)	33.21–33.99
Some College	3352 (33.31)	32.26–34.36	298 (18.01)	15.87–20.15	1205 (15.29)	14.38–16.20	25,796 (38.33)	37.91–38.75
Associate/Bachelors	1958 (16.75)	15.97–17.52	510 (27.13)	24.77–29.48	3017 (35.64)	34.45–36.82	10,956 (12.24)	11.99–12.49
Masters or Higher	1655 (15.94)	15.13–16.75	203 (10.27)	8.75–11.78	1613 (18.43)	17.50–19.36	11,232 (15.83)	15.51–16.15
**Poverty**								
Poor	842 (7.41)	6.82–7.98	239 (13.46)	11.50–15.41	505 (5.74)	5.16–6.32	7929 (10.17)	9.91–10.43
Near Poor	1839 (16.07)	15.27–16.87	393 (22.33)	20.03–24.63	1263 (14.21)	13.36–15.07	13,496 (17.95)	17.63–18.28
Not Poor	8165 (76.52)	75.60–77.45	1111 (64.21)	61.55–66.87	6402 (80.05)	79.06–81.03	54,280 (71.88)	71.49–72.26
**Race/Ethnicity**								
Non-Hispanic White	9246 (83.33)	82.46–84.20	1383 (76.58)	74.09–79.06	7124 (85.71)	84.78–86.65	48,070 (59.96)	59.54–60.38
Non-Hispanic Black	667 (6.41)	5.85–6.96	152 (9.31)	7.61–11.02	442 (5.57)	4.98–6.16	8807 (12.37)	12.08–12.66
Non-Hispanic others	358 (3.83)	3.36–4.29	78 (4.70)	3.43–5.96	241 (3.49)	2.98–4.00	6962 (9.26)	9.02–9.51
Hispanic	575 (6.44)	5.85–7.04	130 (9.41)	7.63–11.20	363 (5.23)	4.62–5.84	11,866 (18.40)	18.06–18.75
**Sexual-Orientation**								
Straight	10,068 (92.41)	91.81–93.02	1602 (91.16)	89.49–92.83	7631 (93.15)	92.50–93.81	67,900 (89.09)	88.82–89.36
Lesbian/Gay	186 (1.54)	1.29–1.80	20 (1.15)	0.52–1.78	145 (1.58)	1.29–1.87	1582 (1.99)	1.87–2.10
Bisexual	104 (1.12)	0.85–1.38	22 (1.90)	0.91–2.89	71 (0.96)	0.70–1.22	1626 (2.50)	2.36–2.65
Not-Sure	488 (4.93)	4.43–5.43	99 (5.79)	4.53–7.06	323 (4.31)	3.77–4.85	4597 (6.42)	6.21–6.23
**Insured**								
Yes	10,671 (97.94)	97.57–98.30	1691 (96.31)	95.13–97.49	8078 (98.61)	98.28–98.94	69,189 (89.79)	89.51–90.07
No	164 (2.06)	1.70–2.43	51 (3.69)	2.51–4.87	86 (1.39)	1.06–1.72	6261 (10.21)	9.93–10.49
**Depressed**								
Yes	1634 (15.37)	14.58–16.16	313 (18.11)	16.05–20.17	1157 (14.49)	13.60–15.38	8578 (10.74)	10.48–10.99
No	9026 (84.63)	83.84–85.42	1393 (81.89)	79.83–83.95	6889 (85.51)	84.62–86.40	65,499 (89.01)	89.01–89.52
**Survey Year**								
2021	3654 (33.36)	32.33–34.38	585 (33.06)	30.48–35.63	2752 (33.35)	32.17–34.52	25,799 (32.99)	32.59–33.38
2022	3430 (32.92)	31.89–33.95	558 (33.81)	31.20–36.43	2570 (32.55)	31.37–33.73	24,184 (33.34)	32.94–33.74
2023	3726 (33.72)	32.70–34.74	600 (33.13)	30.58–35.68	2848 (34.10)	32.93–35.28	25,722 (33.67)	33.27–34.07

^a^ Percentages are weighted (w%) estimates. ^b^ CSs subtypes (SRCs and NSRCs) has 933 missing values. **Abbreviations:** CSs = Cancer Survivors, SRC = Smoking-related cancer, NSRC = Non-Smoking-Related Cancer, CI = Confidence Interval, NHIS = National Health Interview Survey **Note:** Participants with valid responses are included in the analysis, thus may not be reflective of total (N). Also, as per National Cancer Institute: CSs term is intended for adults who have a history of cancer regardless of whether they are free from cancer or not. As per Surgeon General Report (2014), SRCs includes cancers of the bladder, cervix, colon, esophagus, kidney, larynx, liver, lung, mouth, pancreas, rectum, stomach, and throat while NSRCs include cancers of the bone, brain, breast, gallbladder, lymphoma, melanoma, nervous system, ovary, prostate, skin, soft tissue, testis, and uterine

### Prevalence of tobacco product use: exclusive, dual, and poly use

As shown in Fig. 1(a), the TPU were similar among CSs and adults with no cancer history, however, their prevalence varies. CSs had a prevalence twice as high for former-cigarette-only (25.55% vs. 12.13%) users than adults with no cancer history. Compared to adults with no cancer history, they also had a higher prevalence of former dual users [any two products combination] (21.15% vs. 251.55%) and former poly users (11.95% vs. 10.09%). In contrast, CSs compared to their counterpart had lower prevalence for current cigarette-only (5.69% vs. 6.04%), current e-cigarette-only (0.13% vs. 1.43%), current dual [any two products combination] (2.16% vs. 3.85%), and current poly (0.18% vs. 0.61%) users. The *p*-values of < 0.0001 indicates that these TPU also significantly differs between these two populations. 

Similarly, Fig. 1(b) shows TPU within CSs by its subtypes i.e., SRC and NSRC survivors. SRC survivors had higher prevalence of current-cigarette-only (10.22% vs. 4.67%), former-cigarette-only (33.39% vs. 24.71%), current e-cigarette-only (0.15% vs. 0.12%), former e-cigarette-only (1.60% vs. 0.32), current dual use [any two products combination] (3.84% vs. 1.70%), former dual use [any two products combination] (29.10% vs. 24.29%), and current poly (0.15% vs. 0.10%), and former poly (13.11% vs. 11.76%) use compared to NSRC survivors. The *p*-values of < 0.0001 indicates that these TPU also significantly differs between these two populations.

### Tobacco product use pattern—logistic regression analysis

As shown in Tables 2 and 3, compared to adults with no cancer history, CSs were significantly more likely to be former cigarette-only users (aOR: 1.22, 95% CI: 1.13–1.32), former dual [any two products combination] (aOR: 1.21, 95% CI: 1.14–1.29) users, ever-poly-users (aOR: 1.24, 95% CI: 1.15–1.33) and less likely to be ever e-cigarette-only users (aOR: 0.57, 95% CI: 0.39–0.84). However, their current cigarette-only and dual use pattern were not statistically different than the adults with no cancer history. Among CSs, SRC survivors had a significantly higher odds of being ever e-cigarette-only (aOR: 3.41, 95% CI: 1.51–7.70), current-cigarette-only (aOR: 2.26, 95% CI: 1.63–3.11), former-cigarette-only (aOR: 1.85, 95% CI: 1.54–2.23), former dual [any two products combination] (aOR: 1.35, 95% CI: 1.16–1.58), and ever-poly (aOR: 1.32, 95% CI: 1.11–1.57) users compared to NSRC survivors.

**Table 2:** Multinomial regression analysis: tobacco use pattern (exclusive use and dual use) between US CSs (vs. no cancer history), SRCs (vs. NSRCs) survivors, and socio-demographic factors associated with TPU within CSs, NHIS (2021 to 2023)^a^

Characteristics		Exclusive, Dual and Polytobacco Use (aOR, 95% CI)				
Reference: “never users”		Current Cigarette	Former Cigarette	Ever E-Cigarette	Current Dual	Former Dual
**Cancer Status**^b^						
No history of Cancer		Reference	Reference	Reference	Reference	Reference
Cancer Survivors		0.96 (0.83–1.11)	1.22 (1.13–1.32)*	0.57 (0.39–0.84)*	1.09 (0.91–1.31)	1.21 (1.14–1.29)*
**Cancer Subtypes**^3^						
NSRCs		Reference	Reference	Reference	Reference	Reference
SRCs		2.26 (1.63–3.11)*	1.85 (1.54–2.23)*	3.41 (1.51–7.70)*	1.49 (1.00–2.20)	1.35 (1.16–1.58)*
**Age (in years)**						
18–44		Reference	Reference	Reference	Reference	Reference
45–64		1.56 (0.88–2.77)	2.67 (1.71–4.16)*	0.27 (1.71–4.16)*	0.67 (0.41–1.10)	0.72 (0.54–0.95)*
65 and above		0.84 (0.48–1.46)	4.07 (2.66–6.22)*	0.10 (0.04–0.27)*	0.18 (0.11–0.31)*	0.66 (0.51–0.86)*
**Biological Sex**						
Female		Reference	Reference	Reference	Reference	Reference
Male		1.42 (1.06–1.91)*	1.06 (0.91–1.23)	1.02 (0.37–2.79)	4.14 (2.87–5.98)*	5.7 (5.03–6.46)*
**Marital Status**						
Never Married		Reference	Reference	Reference	Reference	Reference
Married		0.82 (0.61–1.09)	0.83 (0.72–0.95)*	0.37 (0.15–0.91)*	0.74 (0.50–1.12)	0.91 (0.80–1.03)
Single After Marriage		1.69 (0.89–3.22)	0.84 (0.53–1.34)	2.28 (0.71–7.28)	3.43 (1.91–6.15)*	1.34 (0.99–1.83)*
**Education level**						
High School or less		Reference	Reference	Reference	Reference	Reference
Some College		0.72 (0.49–1.07)	0.81 (0.66–1.00)*	1.65 (0.40–6.83)	1.34 (0.82–2.17)	1.39 (1.16–1.66)*
Associate/Bachelors		0.34 (0.24–0.48)*	0.58 (0.49–0.70)*	1.37 (0.46–4.06)	0.88 (0.55–1.40)	1.11 (0.95–1.29)*
Master or above		0.12 (0.06–0.22)*	0.49 (0.39–0.60)*	0.44 (0.09–2.07)	0.24 (0.09–0.63)*	0.84 (0.68–1.00)*
**Poverty**						
Poor		Reference	Reference	Reference	Reference	Reference
Near Poor		0.68 (0.43–1.08)	0.98 (0.72–1.33)	1.22 (0.30–4.95)	0.53 (0.31–0.93)*	1.01 (0.75–1.34)
Not Poor		0.51 (0.33–0.79)*	1.09 (0.82–1.46)	1.56 (0.44–5.54)	0.27 (0.16–0.46)*	1.01 (0.78–1.31)
**Race and Ethnicity**^**c**^						
Hispanic		Reference	Reference	Reference	Reference	Reference
Non-Hispanic (all races)		1.72 (0.94–3.14)	1.50 (1.07–2.09)*	2.03 (0.49–8.30)*	3.47 (1.54–7.8)*	2.25 (1.62–3.13)*
**Sexual-Orientation**						
Straight		Reference	Reference	Reference	Reference	Reference
Other genders		1.48 (0.76–2.92)	1.01 (0.70–1.47)	0.62 (0.16–2.38)	1.04 (0.50–2.17)	1.26 (0.90–1.75)
**Insured**						
No		Reference	Reference	Reference	Reference	Reference
Yes		0.49 (0.21–1.15)	1.48 (0.62–3.54)	0.35 (0.07–1.73)	0.32 (0.16–0.65)*	0.74 (0.44–1.24)
**Depressed**						
No		Reference	Reference	Reference	Reference	Reference
Yes		1.45 (1.03–2.05)*	1.33 (1.09–1.61)*	2.19 (0.98–4.93)	2.27 (1.50–3.43)*	1.47 (1.24–1.74)*
**Region**						
Northeast		Reference	Reference	Reference	Reference	Reference
Midwest		1.24 (0.82–1.86)	0.89 (0.72–1.11)	1.52 (0.31–7.48)	1.67 (0.87–3.21)	1.14 (0.94–1.37)
South		0.83 (0.56–1.24)	0.84 (0.69–1.02)	1.17 (0.30–4.56)	1.71 (0.91–3.20)	1.01 (0.84–1.21)
West		0.66 (0.41–1.05)	0.77 (0.62–0.96)*	2.94 (0.77–11.21)	1.10 (0.54–2.25)	1.03 (0.85–1.26)

^**a**^ asterisks (*) indicates *p* < 0.05 and aOR are weighted estimates of the model run only for CSs population

^**b**^ Separate multinomial regression analysis was run for only CSs population to evaluate the pattern of tobacco use among adults with no cancer history vs, CS and its subtype i.e., SRC vs NSRC survivors

^**c**^ The Non-Hispanic races were collapsed together due to very small cell sample size for exclusive use variable to yield a reliable model

*CI = *Confidence Interval, *aOR = *Adjusted Odds Ratio, *NHIS = *National Health Interview Survey, *CS = *Cancer Survivors, *SRC *Smoking-related cancer, *NSRC = *Non-Smoking-Related Cancer, *TPU = *Tobacco Use Patterns

Participants with missing values for tobacco products are excluded from analysis, thus may not be reflective of total (n). The exclusive use categories are mutually exclusive of one another. Also, as per the National Cancer Institute: Cancer Survivors term is intended for adults who have had a history of cancer regardless of whether they are free from cancer or not. As per Surgeon General Report (2014), SRCs includes cancers of the bladder, cervix, colon, esophagus, kidney, larynx, liver, lung, mouth, pancreas, rectum, stomach, and throat while NSRCs include cancers of the bone, brain, breast, gallbladder, lymphoma, melanoma, nervous system, ovary, prostate, skin, soft tissue, testis, and uterine

**Table 3:** Binary regression analysis: tobacco use pattern (exclusive use and dual use) between US CSs (vs. no cancer history), SRCs (vs. NSRCs) survivors, and socio-demographic factors associated with TPU within CSs, NHIS (2021 to 2023)^a^

Characteristics		Poly Use (aOR, 95% CI)
**Reference: ‘never users”**		**Any Polyuse**
**Cancer Status** ^b^		
No history of Cancer		Reference
Cancer Survivors		1.24 (1.15–1.33)*
**Cancer Subtypes**^3^		
NSRCs		Reference
SRCs		1.32 (1.11–1.57)*
**Age (in years)**		
18–44		Reference
45–64		0.56 (0.41–0.75)*
65 and above		0.41 (0.31–0.55)*
**Biological Sex**		
Female		Reference
Male		6.42 (5.52–7.46)*
**Marital Status**		
Never Married		Reference
Married		0.85 (0.73–0.98)*
Single After Marriage		1.76 (1.27–2.44)*
**Education level**		
High School or less		Reference
Some College		1.28 (1.04–1.58)*
Associate/Bachelors		1.03 (0.86–1.22)
Master or above		0.70 (0.56–0.86)*
**Poverty**		
Poor		Reference
Near Poor		0.79 (0.58–1.08)
Not Poor		0.68 (0.52–0.90)*
**Race and Ethnicity **^**c**^		
Non-Hispanic White		Reference
Non-Hispanic Black Non-Hispanic Other Hispanic		0.35 (0.24–0.52)
		0.89 (0.60–1.32)
		0.28 (1.9–0.43)
**Sexual-Orientation**		
Straight		Reference
Other genders		1.19 (0.82–1.74)
**Insured**		
No		Reference
Yes		0.89 (0.51–1.54)
**Depressed**		
No		Reference
Yes		1.62 (1.34–1.96)*
**Region**		
Northeast		Reference
Midwest		1.12 (0.90–1.40)
South		1.01 (0.89–1.35)
West		1.06 (0.85–1.34)

^**a**^ asterisk (*) indicates *p* < 0.05 and aOR are weighted estimates of the model run only for CSs population

^**b**^ Separate multinomial regression analysis was run for only CSs population to evaluate the pattern of tobacco use among adults with no cancer history vs, CS and its subtype i.e., SRC vs NSRC survivors

^**c**^ The Non-Hispanic races were collapsed together due to very small cell sample size for exclusive use variable to yield a reliable model

*CI = *Confidence Interval, *aOR = *Adjusted Odds Ratio, *NHIS = *National Health Interview Survey, *CS = *Cancer Survivors, *SRC = *Smoking-related cancer, *NSRC = *Non-Smoking-Related Cancer, *TPU* = Tobacco Use Patterns

Participants with missing values for tobacco products are excluded from analysis, thus may not be reflective of total (n). The exclusive use categories are mutually exclusive of one another. Also, as per the National Cancer Institute: Cancer Survivors term is intended for adults who have had a history of cancer regardless of whether they are free from cancer or not. As per Surgeon General Report (2014), SRCs includes cancers of the bladder, cervix, colon, esophagus, kidney, larynx, liver, lung, mouth, pancreas, rectum, stomach, and throat while NSRCs include cancers of the bone, brain, breast, gallbladder, lymphoma, melanoma, nervous system, ovary, prostate, skin, soft tissue, testis, and uterine

Table 2 also showed the socio-demographic disparities in exclusive and dual TPU among CSs. Being male (vs. female) significantly increase the likelihood of current-cigarette-only users (aOR:1.42, 95% CI: 1.06–1.91), current dual users (aOR:4.14, 95% CI: 2.87–5.98), and former dual users (aOR:5.7, 95% CI: 5.03–6.46). CSs who were non-Hispanic (vs. Hispanics) also significantly had a higher likelihood of being current dual users (aOR:3.47, 95% CI: 1.54–7.80) and ever e-cigarette users (aOR:2.03, 95% CI: 0.49–8.30). Additionally, CSs who were single after marriage (vs. never married) increase the likelihood of current dual use (aOR:3.43, 95% CI: 1.91–6.15). In contrast, higher educational attainment (vs. lower), being not poor (vs. poor) were significant protective factors towards current cigarette-only use and current dual use. While elder age (≥ 65 vs. 18–44 years) and being married (vs. never married) were significant protective factors against ever e-cigarette only use.

Similarly, Table 3 shows that CSs who were male (vs. female) had a higher likelihood of being ever-poly use (aOR:6.42, 95% CI: 5.52–7.46) and those who were single after marriage (vs. never married) had a higher likelihood of being ever-poly user (aOR:1.76, 95% CI: 1.27–2.44). CSs, who had some college level education (vs. high school or less) had significantly higher likelihood of ever-poly tobacco use (aOR:1.28, 95% CI: 1.04–1.58). Whereas elder age (vs. 18–44 years), being married (vs. never married), masters level education (vs. high school or less) and being not poor (vs. poor) were significant protective factors against ever-poly use behavior.

To explore whether dual use of cigarettes and e-cigarettes might reflect cessation attempts, we examined data from the 2022 NHIS, only survey year which uniquely included questions about use of e-cigarette for cessation. Descriptive analysis (See Online Resource VI) revealed that among small sample of CSs who responded to the questions, 31.28% (n = 22) reported using e-cigarettes during a past-year quit attempt, while 43.74% (n = 13) of former smokers used e-cigarettes to quit completely. However, the limited sample size of CSs restricted further sensitivity and inferential analyses to determine how cessation attempts might influence our observed patterns of dual tobacco use.

## Discussion

This nationally representative study highlights the critical differences in the patterns of exclusive, dual and poly TPU among US adults, with a particular focus on CSs. We found that CSs were more likely to be formerly exclusive, and dual users than current compared to adults with no cancer history, suggesting a greater propensity among them for being involved in multiple TPU in their lifetime. This is reiterated with our finding that CSs (vs. no cancer history) were more likely to be ever-poly users than never users. Although the prevalence of current TPU among CSs is lower than adults with no cancer history, we found a stark disparity in TPU patterns within the CSs population (SRCs vs. NSRCs). This study showed that SRC survivors were twice as likely to be current exclusive cigarette users than NSRC survivors aligning with previous studies that showed that SRC survivors were likely to continue smoking post-diagnosis than others [15, 35, 36]. This suggests that, while cessation effectiveness among CSs and adults with no cancer history appears comparable, it remains important to assess whether survivors of SRCs and NSRCs benefits equally.

In addition, our study found that the survivors of SRCs (vs. NSRC) were also more likely to be ever-poly users and the most prevalent dual use combination among them are “cigarette with e-cigarettes” followed by “cigarettes with cigar.” This varied TPU patterns highlighted a critical aspect of tobacco/nicotine use behavior among CSs, that they may not only use cigarette or a single tobacco product but instead engage in a pattern of switching or combining different tobacco products, which is consistent with the recent clinical study [20]. This behavior could be rooted in the tobacco/nicotine dependency and related addiction complexities among CSs [37]. The dual use of “cigarette with e-cigarettes” among CSs is well-studied and is often attributed to the perceived benefit as a cessation tool and less harmful compared to cigarettes by the users. However, a recent meta-analysis indicated that compared with exclusive cigarette users, dual users had higher odds (20–40%) of tobacco-associated diseases (i.e., cardiovascular, stroke, asthma etc.) and compared with never users they had up to threefold increased risk [38]. Despite this, nearly 72% of dual users never discussed their e-cigarette use with their oncology providers, underscoring the commination gap in oncology clinics [12]. Given the inconclusive evidence regarding the effectiveness of e-cigarettes as cessation tools, these dual users not only have an exposure to the carcinogens in cigarettes but also to the toxic and potentially lethal constituents of e-cigarettes, which may compound harm [7, 39, 40]. It is important to note that distinguishing between e-cigarette use for cessation attempts versus sustained poly-tobacco use remains a complex question in this population. Our analysis using 2022 NHIS data, the only year that included questions about e-cigarette use for cessation, revealed limited sample sizes (n = 22–44, depending on the specific cessation question) that prevented comprehensive analysis of cessation motivations. Future research with larger samples should specifically examine the motivations and contexts of e-cigarette use among CSs to better understand the interplay between harm reduction/cessation efforts and recreational poly-tobacco use patterns.

While the use of “cigarette with e-cigarette” is well documented, the motivations and perceptions behind combination use like “cigar with cigarette” still remain unclear and poorly understood [4, 8, 9]. Therefore, understanding perceptions is essential because misconceptions about product safety lead to harmful dual use patterns. CSs who are dual users may believe that certain combinations aid their cessation efforts or are less harmful, but evidence remain inconclusive and dual use has been reported to increase health risks [38]. Additionally, perceptions such as personal beliefs, social and psychological factors (e.g., stress and social acceptability) affect treatment adherence; survivors who prefer e-cigarettes as cessation tool find them more satisfying, less addictive, and more effective in reducing cancer-related stress than FDA-approved nicotine replacement therapy [12]. Hence, potentially hindering the overall cessation effectiveness among this vulnerable group. This illustrates that without an understanding of CSs’ perceptions behind TPU patterns, clinical efforts will remain generic. Perception-based research is fundamental to developing targeted and culturally appropriate cessation strategies that align with survivors' actual beliefs and decision-making processes.

Apart from evaluating perceptions behind varied TPU patterns among CSs our findings showed that certain socio-demographics are more susceptible to exclusive, dual, or poly TPU which includes being male, single after marriage, or having lower educational attainment while belong to elder age groups may decrease the odds of being dual or ever-poly users. These socio-demographic differences in TPU pattern are consistent with other previous studies [4, 41, 42]. We also found that CSs who were depressed showed higher susceptibility across each measure of TUPs except for ever e-cigarette only use. Therefore, our study highlights the importance of identifying the multifaceted nature of cessation barriers that exists at the patient, provider, and system levels [43–45]. These include patient-level issues like readiness to quit and misconceptions about health risks, providers-level issues like limited counseling due to time-constraints and inconsistent assessment of tobacco use and systemic challenges like weak referral pathways [43–45]. To close these gaps, existing cessation programs can be enhanced by offering oncologists targeted training focused on dual and poly use patterns, standardizing comprehensive tobacco-use assessments in electronic health records that include all tobacco products, and strengthening referral systems to ensure timely access to evidence-based support designed for complex use patterns.

At the clinical level, we recommend moving beyond one-size-fits-all approaches. CSs should be guided toward evidence-based cessation strategies: pharmacological therapies (e.g., NRT, bupropion, varenicline) [46] combined with behavioral counseling specifically tailored for dual/poly users. Providers should initiate discussions about suspected or potential use of other tobacco products at each follow-up. For dual users, especially those who use a combination of cigarettes and e-cigarettes, existing cessation protocols should be modified to include gradual reduction strategies and clear communication about both the risks and limitations of e-cigarettes as tools for quitting. Occasional biomarker screening can help address self-report bias and track cessation progress across multiple products. Lastly, at the Patient/Family Level, providers should be trained to discuss nicotine concentration, delivery methods, and motivations for product use to customize interventions to individual needs. Since smoking often occurs in social groups, existing family-based cessation programs should be adapted to manage the complexities of dual/poly use, including education on the health risks of using multiple products and support strategies for families dealing with complex tobacco use patterns [47, 48]. Moreover, cessation strategies should address the varied dependencies and behavioral triggers associated with each tobacco product. Exclusive users can benefit from standard evidence-based protocols tailored to their specific level of dependence [49]. In contrast, dual and poly users requires more intensive and complex approaches to manage addiction arising from multiple sources. For example, dual users of cigarettes and e-cigarettes often hold misconceptions that e-cigarettes are safer and effective transition tools; thus a staged approach may be implemented i.e., eliminating cigarettes first, followed by e-cigarette use through FDA approved methods [50]. Poly users require highly individualized strategies, such as comprehensive behavioral mapping and intensive counseling, to address TPU patterns across various contexts (e.g., social vs. private settings).

Lastly, although this study has highlighted critical aspects of TPU patterns among CSs, it has some limitations. Firstly, its cross-sectional nature precludes causality and self-reported data on smoking behaviors may have introduced social desirability bias. Furthermore, survival bias might lead to an underrepresentation of CSs particularly SRC survivors due to higher mortality rates among them than NSRC. Thus, it may not accurately reflect the true smoking patterns among this vulnerable population. Despite this, our study benefits from the use of recently available data, allowing for more accurate estimates and unlike previous studies, our research included exclusive tobacco use alongside other TUPs to provide a comprehensive picture of smoking behaviors among CSs and its subtypes. Overall, our results underscore the critical need for timely and targeted smoking cessation interventions within oncology setting by understanding the perception behind various TUPs, i.e., dual and poly. Thus, enhancing survivorship care and overall long-term health.

## Conclusion

Disparities in TPU among CSs particularly between SRC survivors and NSRC survivors calls for the need for proactive cessation support. Healthcare providers, mainly oncologists, should address the TPU systematically in their routine oncology visits through integrated and tailored counselling strategies early in survivorship care. These strategies must account for patients’ former and current TPU, their perceptions behind these patterns, and socio-demographic barriers to quitting. Furthermore, longitudinal and clinical research are needed to better understand perceptions underlying various TPU, particularly dual/poly-tobacco use, to develop more effective evidence-based interventions.

## Supplementary Information

Below is the link to the electronic supplementary material.

ESM 1 (DOCX 184 KB)

## Data Availability

Data can be accessed at the Centers for Disease Control and Prevention (CDC) website: [https://www.cdc.gov/nchs/nhis/index.htm](https://www.cdc.gov/nchs/nhis/index.htm).
