# Supplementary material for: Patterns of exclusive, dual and poly tobacco products use among cancer survivors in the United States
Source: J Cancer Surviv. 2025 Dec 16;20(5):2520–31. doi: 10.1007/s11764-025-01918-x (PMC12754734; doi:10.1007/s11764-025-01918-x)
Supplement: Supplementary file 1 [file 11764_2025_1918_MOESM1_ESM.docx]

**Patterns of Exclusive, Dual and Poly-Tobacco Use Among Cancer Survivors in the United States**

I.**Online Resource I: Study Sample Flow-Chart**

**Online Resource I. Study Participants flow chart.**

II. **Online Resource II: Presented as Figure 1(a)**

**Online Resource II.**
Prevalence of Exclusive, Dual and Poly tobacco use among the U.S. adults with no Cancer History and CSs, NHIS 2021 to 2023.^1^

| **Tobacco Use Pattern** | **Cancer Survivors (N= 9,913)** | | **No Cancer History (N = 75,705)** | | **p-Value** |
| --- | --- | --- | --- | --- | --- |
|  | **N (w%)** | **95% CI** | **N (w%)** | **95% CI** |  |
| **Exclusive Use** | | | | |  |
| Current-Cigarette | 359 (5.69) | 5.03 – 6.36 | 3056 (6.04) | 5.79 – 6.29 | **<0.0001** |
| Former-Cigarette | 1808(25.55) | 24.36 – 26.73 | 7042 (12.13) | 11.81 – 12.45 |  |
| Current E-Cigarette Only | 9 (0.13) | 0.04 – 0.22 | 448 (1.43) | 1.27 – 1.58 |  |
| Former E-Cigarette Only | 34 (0.63) | 0.38 – 0.89 | 1242 (3.48) | 3.25 – 3.70 |  |
| Never | 4499 (68) | 66.71 – 69.28 | 35131 (76.93) | 76.47 – 77.37 |  |
| **Dual Use** | |  |  |  |  |
| **Current^2^** | 196 (2.16) | 1.82 – 2.50 | 2568 (3.85) | 3.68 – 4.02 | **<0.0001** |
| Cig + E-Cig | 84 (0.97) | 0.74 – 1.20 | 1090 (1.65) | 1.53 – 1.76 |  |
| Cig + Cigar | 65 (0.68) | 0.49 – 0.87 | 606 (0.86) | 0.77 – 0.94 |  |
| **Former^3^** | 2627 (25.12) | 24.17 – 26.07 | 16139 (21.15) | 20.80 – 21.49 |  |
| Cig + E-Cig | 342 (3.67) | 3.23 – 4.11 | 3135 (4.24) | 4.07 – 4.41 |  |
| Cig + Cigar | 1392 (12.46) | 11.78 – 13.16 | 5571 (6.78) | 6,56 – 6.96 |  |
| **Never** | 7771 (72.72) | 71.74 – 72.70 | 54714 (75) | 74.64 – 75.37 |  |
| **Poly Use** |  |  |  |  |  |
| Current | 17 (0.18) | 0.09 – 0.28 | 390 (0.61) | 0.54 – 0.68 | 0.211 |
| Former | 1273 (11.95) | 11.24 – 12.65 | 7951 (10.09) | 9.84 – 10.34 |  |
| Never | 9556 (87.87) | 87.16 – 88.58 | 67364 (89.30) | 89.05 – 89.56 |  |

^1^ Percentages are weighted (w) estimates and the exclusive use categories are mutually exclusive of one another.
**^2,3^** Dual-use behavior which was most prevalent with adequate sample size was added in the main table. Rest can be found in the supplemental table (A).
**Abbreviations:** CI = Confidence Interval. Cancer Survivors = CSs, NHIS = National Health Interview Survey. Cigarettes, E-Cig = E-cigarettes, TUP = Tobacco Use Pattern
**Notes: (1)** Analysis included only participants who met their respective TUP variable criteria with valid responses; hence sample sizes (n) may differ from total population (N). **(2)** p-values test whether tobacco patterns significantly differs between two populations, while N (w%) are respective to each group separately. **(3)** As per National Cancer Institute: Cancer Survivors term is intended for adults who have a history of cancer regardless of whether they are free from cancer or not. **(4)** As per Surgeon General Report (2014), SRCs includes cancers of the bladder, cervix, colon, esophagus, kidney, larynx, liver, lung, mouth, pancreas, rectum, stomach, and throat while NSRCs include cancers of the bone, brain, breast, gallbladder, lymphoma, melanoma, nervous system, ovary, prostate, skin, soft tissue, testis, and uterine.

III. **Online Resource III: Presented as Figure 1(b)**

**Online Resource III.**
Prevalence of Exclusive, Dual and Poly tobacco use among CSs subtypes, NHIS, 2021 to 2023.^1^

| **Tobacco Use** | **SRC Survivors (N= 1,743)** | | **NSRC Survivors (N= 8,170)** | | **p-Value** |
| --- | --- | --- | --- | --- | --- |
|  | **N (w%)** | **95% CI** | **N (w%)** | **95% CI** |  |
| **Exclusive Users** | | | | | |
| Current-Cigarette | 96 (10.22) | 7.98 – 12.46 | 228 (4.67) | 3.98 – 5.36 | **<0.0001** |
| Former-Cigarette | 323 (33.39) | 29.91 – 36.87 | 1353 (24.70) | 23.38 – 26.03 |  |
| Current E-Cigarette Only | 2 (0.15) | 0.00 – 0.37 | 6 (0.12) | 0.02 – 0.22 |  |
| Former E-Cigarette Only | 12 (1.60) | 0.56 – 2.64 | 17 (0.32) | 0.15 – 0.49 |  |
| Never | 531 (54.64) | 50.93 – 58.33 | 3561 (70.19) | 68.76 – 71.61 |  |
| **Dual Users** |  |  |  |  |  |
| **Current** | 60 (3.84) | 2.79 – 4.89 | 117 (1.70) | 1.35 – 2.06 | **<0.0001** |
| Cig + E-Cig (w%) | 31 (1.96) | 1.21 – 2.71 | 46 (0.73) | 0.50 – 0.96 |  |
| Cig + Cigar (w%) | 21 (1.24) | 0.66 – 1.81 | 38 (0.53) | 0.33 – 0.73 |  |
| **Former** | 489 (29.10) | 26.57 – 31.62 | 1926 (24.29) | 23.21 – 25.37 |  |
| Cig + E-Cig (w%) | 78 (4.83) | 3.63 – 6.03 | 224 (3.18) | 2.71 – 3.65 |  |
| Cig + Cigar (w%) | 245 (13.88) | 12.03 – 15.71 | 1058 (12.59) | 11.78 – 13.40 |  |
| **Never** | 1140 (67.06) | 64.45 – 69.68 | 5965 (74.01) | 72.90 – 75.11 |  |
| **Ever Poly** |  |  |  |  |  |
| Current | 3 (0.15) | 0.01 – 0.31 | 9 (0.10) | 0.03 – 0.18 | **<0.0001** |
| Former | 234 (13.11) | 11.33 – 14.89 | 941 (11.76) | 10.95 – 12.56 |  |
| Never | 1506 (86.74) | 84.96 – 88.53 | 7220 (88.14) | 87.33 – 88.95 |  |

^1^ Percentages are weighted (w) estimates and the exclusive use categories are mutually exclusive of one another.
**^2,3^** Dual-use behavior which was most prevalent with adequate sample size was added in the main table. Rest can be found in the supplemental table (A).
**Abbreviations:** CI = Confidence Interval. Cancer Survivors = CSs, NHIS = National Health Interview Survey. Cigarettes, E-Cig = E-cigarettes, TUP = Tobacco Use Pattern
**Notes: (1)** Analysis included only participants who met their respective TUP variable criteria with valid responses; hence sample sizes (n) may differ from total population (N). **(2)** p-values test whether tobacco patterns significantly differs between two populations, while N (w%) are respective to each group separately. **(3)** As per National Cancer Institute: Cancer Survivors term is intended for adults who have a history of cancer regardless of whether they are free from cancer or not. **(4)** As per Surgeon General Report (2014), SRCs includes cancers of the bladder, cervix, colon, esophagus, kidney, larynx, liver, lung, mouth, pancreas, rectum, stomach, and throat while NSRCs include cancers of the bone, brain, breast, gallbladder, lymphoma, melanoma, nervous system, ovary, prostate, skin, soft tissue, testis, and uterine.

IV. **Online Resource IV: All Dual-Use Combinations used to derive dual-use variable**

**Online Resource IV.**
Prevalence of Dual tobacco use by CSs status, NHIS, 2021 to 2023.^1^

| **Tobacco Use** | **Cancer Survivors (N= 10,846)** | | **No Cancer History (N = 75,705)** | | **p-Value** |
| --- | --- | --- | --- | --- | --- |
|  | **N (w%)** | **95% CI** | **N (w%)** | **95% CI** |  |
| **Dual Users** |  |  |  |  | **<0.0001** |
| **Current** | **196 (2.16)** | **1.82 – 2.50** | **2568 (3.85)** | **3.68 – 4.02** |  |
| Cig + E-Cig | 84 (0.97) | 0.74 – 1.20 | 1090 (1.65) | 1.53 – 1.76 |  |
| Cig + Cigar | 65 (0.68) | 0.49 – 0.87 | 606 (0.86) | 0.77 – 0.94 |  |
| Cig + Pipe | 08 (0.08) | 0.02 – 0.14 | 81 (0.10) | 0.08 – 0.13 |  |
| Cig + SMLT | 13 (0.16) | 0.06 – 0.27 | 172 (0.23) | 0.19 – 0.27 |  |
| E-Cig + Cigar | 02 (0.03) | 0.00 – 0.09 | 233 (0.40) | 0.34 – 0.46 |  |
| E-Cig + Pipe | 03 (0.03) | 0.00 – 0.07 | 60 (0.10) | 0.07 – 0.14 |  |
| E-Cig + SMLT | 06 (0.07) | 0.01 – 0.14 | 85 (0.15) | 0.11 – 0.18 |  |
| Cigar + Pipe | 06 (0.05) | 0.01 – 0.09 | 111 (0.17) | 0.13 – 0.21 |  |
| Cigar + SMLT | 08 (0.08) | 0.02 – 0.14 | 118 (0.18) | 0.14 – 0.22 |  |
| Pipe + SMLT | 01 (0.00) | 0.00 – 0.01 | 12 (0.02) | 0.01 – 0.03 |  |
| **Former** | **2627 (25.12)** | **24.17 – 26.07** | **16139 (21.15)** | **20.80 – 21.49** |  |
| Cig + E-Cig | 342 (3.67) | 3.23 – 4.11 | 3135 (4.24) | 4.07 – 4.41 |  |
| Cig + Cigar | 1392 (12.46) | 11.78 – 13.16 | 5571 (6.78) | 6,56 – 6.96 |  |
| Cig + Pipe | 202 (1.78) | 1.51 – 2.06 | 589 (0.68) | 0.61 – 0.74 |  |
| Cig + SMLT | 49 (0.50) | 0.34 – 0.67 | 303 (0.41) | 0.35 – 0.46 |  |
| E-Cig + Cigar | 225 (2.57) | 2.18 – 2.96 | 3207 (4.63) | 4.45 – 4.81 |  |
| E-Cig + Pipe | 21 (0.25) | 0.12 – 0.38 | 507 (0.78) | 0.70 – 0.86 |  |
| E-Cig + SMLT | 10 (0.14) | 0.04 – 0.23 | 129 (0.22) | 0.17 – 0.26 |  |
| Cigar + Pipe | 280 (2.54) | 2.21 – 2.87 | 1828 (2.23) | 2.11 – 2.34 |  |
| Cigar + SMLT | 98 (1.16) | 0.90 – 1.41 | 785 (1.09) | 1.00 – 1.17 |  |
| Pipe + SMLT | 08 (0.06) | 0.01 – 0.11 | 85 (0.12) | 0.09 – 0.15 |  |
| **Never** | **7771 (72.72)** | **71.74 – 72.70** | **54714 (75)** | **74.64 – 75.37** |  |

^1^ Percentages are weighed (w) estimates.
**Abbreviations:** CI = Confidence Interval. NHIS = National Health Interview Survey, Cig = Cigarettes, E-Cig = E-cigarettes. SMLT= smokeless tobacco **Note:** Participants with missing values for tobacco products are excluded from analysis, thus may not be reflective of total (n). Also, as per the National Cancer Institute: Cancer Survivors term is intended for adults who have had a history of cancer regardless of whether they are free from cancer or not.
As per Surgeon General Report (2014), SRCs includes cancers of the bladder, cervix, colon, esophagus, kidney, larynx, liver, lung, mouth, pancreas, rectum, stomach, and throat while NSRCs include cancers of the bone, brain, breast, gallbladder, lymphoma, melanoma, nervous system, ovary, prostate, skin, soft tissue, testis, and uterine.
**Note:** The table is provided to maintain transparency and all of the above results are not mentioned in main results due to sample size and reliability.

V. **Online Resource V: Table D: All Dual-Use Combinations used to derive dual-use variable**

**Online Resource V.**
Prevalence of Dual tobacco use among CSs subtypes, NHIS, 2021 to 2023.^1^

| **Tobacco Use** | **SRC Survivors (N= 1,743)** | | **NSRC Survivors (N= 8,170)** | | **p-Value** |
| --- | --- | --- | --- | --- | --- |
|  | **N (w%)** | **95% CI** | **N (w%)** | **95% CI** |  |
| **Dual Users** |  |  |  |  | **<0.0001** |
| **Current** | **60 (3.84)** | **2.79 – 4.89** | **117 (1.70)** | **1.35 – 2.06** |  |
| Cig + E-Cig | 31 (1.96) | 1.21 – 2.71 | 46 (0.73) | 0.50 – 0.96 |  |
| Cig + Cigar | 21 (1.24) | 0.66 – 1.81 | 38 (0.53) | 0.33 – 0.73 |  |
| Cig + Pipe | 02 (0.21) | 0.00 – 0.51 | 06 (0.05) | 0.01 – 0.10 |  |
| Cig + SMLT | 03 (0.21) | 0.00 – 0.48 | 07 (0.12) | 0.01 – 0.23 |  |
| E-Cig + Cigar | 01 (0.11) | 0.00 – 0.32 | 02 (0.05) | 0.00 – 0.12 |  |
| E-Cig + Pipe | 02 (0.12) | 0.00 – 0.30 | 03 (0.04) | 0.00 – 0.09 |  |
| E-Cig + SMLT | 0 |  | 02 (0.03) | 0.00 – 0.08 |  |
| Cigar + Pipe | 0 |  | 04 (0.04) | 0.00 – 0.07 |  |
| Cigar + SMLT | 0 |  | 08 (0.11) | 0.03 – 0.19 |  |
| Pipe + SMLT | 0 |  | 01 (0.00) | 0.00 – 0.01 |  |
| **Former** | **489 (29.10)** | **26.57 – 31.62** | **1926 (24.29)** | **23.21 – 25.37** |  |
| Cig + E-Cig | 78 (4.83) | 3.63 – 6.03 | 224 (3.18) | 2.71 – 3.65 |  |
| Cig + Cigar | 245 (13.88) | 12.03 – 15.71 | 1058 (12.59) | 11.78 – 13.40 |  |
| Cig + Pipe | 30 (1.75) | 1.06 – 2.44 | 151 (1.73) | 1.42 – 2.04 |  |
| Cig + SMLT | 10 (0.56) | 0.13 – 0.98 | 36 (0.52) | 0.32 – 0.71 |  |
| E-Cig + Cigar | 65 (4.33) | 3.06 – 5.60 | 135 (2.07) | 1.68 – 2.47 |  |
| E-Cig + Pipe | 03 (0.25) | 0.00 – 0.62 | 17 (0.27) | 0.12 – 0.41 |  |
| E-Cig + SMLT | 04 (0.27) | 0.00 – 0.54 | 04 (0.08) | 0.00 – 0.16 |  |
| Cigar + Pipe | 42 (2.25) | 1.50 – 3.00 | 216 (2.59) | 2.21 – 2.97 |  |
| Cigar + SMLT | 11 (0.96) | 0.29 – 1.62 | 78 (1.19) | 0.90 – 1.48 |  |
| Pipe + SMLT | 01 (0.02) | 0.00 – 0.07 | 07 (0.08) | 0.01 – 0.14 |  |
| **Never** | **1140 (67.06)** | **64.45 – 69.68** | **5965 (74.01)** | **72.90 – 75.11** |  |

^1^ Percentages are weighed (w) estimates.
**Abbreviations:** CI = Confidence Interval. NHIS = National Health Interview Survey, Cig = Cigarettes, E-Cig = E-cigarettes. SMLT= smokeless tobacco **Note:** Participants with missing values for tobacco products are excluded from analysis, thus may not be reflective of total (n). Also, as per the National Cancer Institute: Cancer Survivors term is intended for adults who have had a history of cancer regardless of whether they are free from cancer or not. As per Surgeon General Report (2014), SRCs includes cancers of the bladder, cervix, colon, esophagus, kidney, larynx, liver, lung, mouth, pancreas, rectum, stomach, and throat while NSRCs include cancers of the bone, brain, breast, gallbladder, lymphoma, melanoma, nervous system, ovary, prostate, skin, soft tissue, testis, and uterine.
**Note:** The table is provided to maintain transparency and all of the above results are not mentioned in main results due to sample size and reliability.

I. **Online Resource VI: Table E: Sensitivity Analysis**

**Online Resource VI.**
E-cigarette Use as Cessation tool among the U.S. adults with no Cancer History and CSs, NHIS 2022 (N=27,614).^1^

| **Tobacco Use** | **Cancer Survivors (N = 3430)** | | **No Cancer History (N= 24,184)** | | **p-Value** |
| --- | --- | --- | --- | --- | --- |
|  | **N (w%)** | **95% CI** | **N (w%)** | **95% CI** |  |
| **Stopped Smoking Completely by Switching to R-cigarettes** | | | | |  |
| Yes | 13 (31.28%) | 21.93 – 65.54 | 263 (61.44%) | 55.38 – 66.49 | 0.239 |
| No | 14 (56.26%) | 34.46 – 78.07 | 193 (38.56%) | 33.51 – 43.62 |  |
| **Attempt to Cessation by switching to E-cigarettes**^2^ | | | | | |
| Yes | 22 (31.28%) | 18.53 – 44.04 | 241(39.56%) | 32.23 – 40.61 | 0.094 |
| No | 44 (68.71%) | 55.96 – 81.47 | 402 (60.44%) | 55.96 – 64.91 |  |

^1^ Only the 2022 NHIS survey measured the cessation/cessation attempt using E-cigarettes as a cessation tool. 2021 and 2023 does not include these measures
^2^ Attempt to cessation was measured for the past 12 months.
